# Efficacy of a Theory-Based Cognitive Behavioral Technique App-Based Intervention for Patients With Insomnia: Randomized Controlled Trial

**DOI:** 10.2196/15841

**Published:** 2020-04-01

**Authors:** Nilofar Rajabi Majd, Anders Broström, Martin Ulander, Chung-Ying Lin, Mark D Griffiths, Vida Imani, Daniel Kwasi Ahorsu, Maurice M Ohayon, Amir H Pakpour

**Affiliations:** 1 Social Determinants of Health Research Center Research Institute for Prevention of Non-Communicable Diseases Qazvin University of Medical Sciences Qazvin Iran; 2 Department of Nursing School of Health and Welfare Jönköping University Jönköping Sweden; 3 Department of Clinical Neurophysiology Linköping University Hospital Linköping Sweden; 4 Department of Rehabilitation Sciences Hong Kong Polytechnic University Hung Hom China (Hong Kong); 5 International Gaming Research Unit Psychology Department Nottingham Trent University Nottingham United Kingdom; 6 Pediatric Health Research Center Tabriz University of Medical Sciences Tabriz Iran; 7 Division of Public Mental Health and Population Sciences Stanford Sleep Epidemiology Research Center Stanford University Palo Alto, CA United States

**Keywords:** app-based intervention, cognitive behavioral therapy, insomnia, sleep hygiene, theory of planned behavior

## Abstract

**Background:**

Sleep hygiene is important for maintaining good sleep and reducing insomnia.

**Objective:**

This study examined the long-term efficacy of a theory-based app (including cognitive behavioral therapy [CBT], theory of planned behavior [TPB], health action process approach [HAPA], and control theory [CT]) on sleep hygiene among insomnia patients.

**Methods:**

The study was a 2-arm single-blind parallel-group randomized controlled trial (RCT). Insomnia patients were randomly assigned to a treatment group that used an app for 6 weeks (ie, CBT for insomnia [CBT-I], n=156) or a control group that received only patient education (PE, n=156) through the app. Outcomes were assessed at baseline and 1 month, 3 months, and 6 months postintervention. Primary outcomes were sleep hygiene, insomnia, and sleep quality. Secondary outcomes included attitudes toward sleep hygiene behavior, perceived behavioral control, behavioral intention, action and coping planning, self-monitoring, behavioral automaticity, and anxiety and depression. Linear mixed models were used to evaluate the magnitude of changes in outcomes between the two groups and across time.

**Results:**

Sleep hygiene was improved in the CBT-I group compared with the PE group (*P*=.02 at 1 month, *P*=.04 at 3 months, and *P*=.02 at 6 months) as were sleep quality and severity of insomnia. Mediation analyses suggested that perceived behavioral control on sleep hygiene as specified by TPB along with self-regulatory processes from HAPA and CT mediated the effect of the intervention on outcomes.

**Conclusions:**

Health care providers might consider using a CBT-I app to improve sleep among insomnia patients.

**Trial Registration:**

ClinicalTrials.gov NCT03605732; https://clinicaltrials.gov/ct2/show/NCT03605732

## Introduction

### Sleep and Insomnia

Inadequate sleep and sleep disorders are among the most frequent problems worldwide [1]. Insomnia is the most common sleep disorder affecting approximately one-third of the general population [2]. It can have negative consequences on one or several spheres in daily life: psychosocial (eg, depression, daytime dysfunction, reduced quality of life), occupational functioning (eg, job absence, reduced ability to do tasks, poor job satisfaction, and inappropriate decisions and choices), or elevated burden to society (eg, increased health costs, reduced job productivity) [3-5]. Insomnia is described as a dissatisfying sleep associated with difficulty initiating or maintaining sleep or early morning awakening despite good opportunities for sleep leading to various daytime symptoms.

Introducing good sleep hygiene practices is often one of the first steps in treating insomnia. Sleep hygiene refers to a variety of behaviors that promote sleep quality [6]. Sleep promoting behaviors include avoiding going to bed hungry or thirsty, avoiding stress and anxiety, avoiding physical activity before going to bed, preparing a bedroom that provides a relaxed environment, and limiting activities in the bedroom to sleep and sexual activities [7]. However, behavioral modifications to improve sleep quality can be hard to achieve; many patients with insomnia report they have tried to modify their poor sleep habits without any effect. Dysfunctional or unrealistic sleep expectations and excessive worrying over sleep loss appear to contribute to poor sleep hygiene. For instance, a Japanese study found that sleep hygiene behaviors are confounded by sleep beliefs [8]. Additionally, due to the rise of mobile technologies, many individuals now use electronic devices in bed, and this may restrict individuals with insomnia from practicing good sleep hygiene (eg, being too excited to sleep due to the use of media) [9]. Consequently, interventions are needed to help individuals with insomnia actually practice sleep hygiene.

Simply providing intervention on sleep hygiene behaviors for individuals with insomnia is insufficient. A review paper of qualitative studies showed that individuals suffering from insomnia observe the inefficiency of sleep hygiene education delivered by health care providers. In particular, health care providers are found to have limited knowledge in sleep hygiene (ie, they only know a few basic principles), and most providers are unable to incorporate theoretical models or techniques to deliver sleep hygiene [10]. Therefore, interventions concerning sleep hygiene behaviors should incorporate robust and effective theoretical models. Indeed, in a systematic review and meta-analysis, Webb et al [11] found that using theories as a framework in designing online interventions led to a substantial effect on outcome variables. Theoretical models can help to select the components of the intervention and help in evaluation of the intervention’s impact [12-22]. By designing interventions based on empirically derived theoretical principles, researchers can identify the most powerful determinants of a given construct [23]. Despite evidence-based recommendations that support the utility of theory-based approaches for designing interventions [21], very few studies have considered this aspect [22,24]. Therefore, studies using theory to support treatment efficacy are much needed.

There are four empirically validated theoretical models (cognitive behavioral therapy [CBT], theory of planned behavior [TPB], health action process approach [HAPA], and control theory [CT]) that may assist individuals with insomnia in engaging good sleep hygiene.

### Cognitive Behavioral Therapy

CBT focuses on the effect of individuals’ beliefs, thoughts, and attitudes on their feelings and behaviors. The purpose of CBT is to educate individuals on how to proactively deal with problems or events during their lives [25]. CBT for insomnia (CBT-I) relies on the general principles of CBT and is designed to relieve insomnia symptoms [26]. The efficacy of CBT-I has been demonstrated in studies [27,28], and a randomized controlled trial (RCT) found that CBT-I outperformed medication in the long run [28]. The principles of CBT are presented to insomnia patients as a set of scientific methods with assessed efficacy [29]. The principles of CBT-I include behavioral modifications such as stimulus control, sleep restriction, sleep hygiene, relaxation training, and cognitive therapy. The goal is to adjust sleeping habits using cognitive strategies to improve thoughts, feelings, and expectations concerning sleep [30,31]. CBT-I can be held in group, individual face-to-face, or online (eg, app-based) sessions based on the therapist’s priority, patient’s progress, or both [32]. Although the efficacy of online CBT-I has been well established for general populations [25], there is a lack of understanding of how the efficacy of online CBT-I is explained by psychological mechanisms [33,34]. Therefore, this study incorporated CBT-I techniques using an app and investigated the underlying psychological mechanisms in the effectiveness of CBT-I, for which evidence has been extensively presented in the literature [26,28-33].

### Theory of Planned Behavior

TPB is one of the most widely applied sociocognitive theories. According to TPB, behavioral intention—an individual’s expression of their decisions to perform or not perform a specific behavior—is the most proximal determinant of an individual’s behavior. Behavioral intention, in turn, is determined by three constructs: attitudes toward the behavior (ie, whether an individual prefers or values the specific behavior), subjective norms (ie, the opinions of the individual’s significant others on the specific behavior), and perceived behavioral control (ie, how confident the individual feels in performing the specific behavior) [35]. The TPB has been used for understanding a variety of behaviors including sleep hygiene behaviors [7,36-38]. Based on TPB, health care providers may try to improve the attitude, subjective norm, and perceived behavioral control of individuals on their sleep hygiene behaviors. Subsequently, the individual may have elevated intention to practice good sleep hygiene.

### Health Action Process Approach

Despite the success of TPB, there is evidence that intentions do not necessarily translate into actual behavior (ie, there is an intention-behavior gap) [39]. For this reason, HAPA proposes a series of volitional, or postintentional, processes that aim to bridge the intention-behavior gap. HAPA focuses more on postintentional processes than TPB. The volitional phase specifies both action planning and coping planning as mediators of the intention-behavior relationship. Action planning indicates how individuals move their intention into action (in this case, sleep hygiene behaviors). More specifically, individuals make plans to practice their sleep hygiene behavior. Coping planning indicates how individuals design any plan to overcome the possible obstacles in the intended behavior (again in this case, sleep hygiene). More specifically, individuals make plans to avoid any potential obstructions that hinder their sleep hygiene behaviors [38]. Using action and coping planning, individuals can better equip their intention to initiate sleep hygiene behaviors. Health care providers may assist individuals with insomnia to facilitate action and coping plans to ensure that individuals’ sleep hygiene behaviors can be implemented and actualized.

### Control Theory

CT explains self-regulation systems, which are critical elements in analyzing human behaviors. The fundamental idea of CT is the negative feedback loop, and a behavior is performed when the discrepancy between current state (eg, drink a cup of tea before sleep) and reference value (eg, should not drink caffeine prior to sleep) is observed by individuals [40]. In order to assess the discrepancy, the technique of self-monitoring can be incorporated to the theoretical models mentioned above (CBT, TPB, and HAPA) to enhance the behavioral intention in performing sleep hygiene behaviors.

### Related Work on App

The insomnia intervention was delivered via a smartphone app to make it available and accessible to meet population needs; app-based interventions overcome some of the pragmatic problems of the face-to-face therapy, including time limitations and high costs [41,42]. Indeed, app-based programs have been developed with highly personalized, tailored, and fully automated features [43-45]. In relation to exposure of in-person contact, a CBT-I app can be used as supplementary support to in-person therapy or as a primary intervention for insomnia through fully automated CBT-I (ie, the therapeutic program can be offered without any human support) [45]. The app designed in this study is similar to the CBT-i Coach [46]. More specifically, the app used in this study was designed to increase patients’ knowledge and skills using CBT-I (for detailed information please refer to the app overview section in the Methods). Both apps attempt to reduce insomnia symptoms. Although the CBT-i Coach includes sleep hygiene recommendations in the session, the app used in our study directly addresses practicing sleep hygiene behaviors. Specifically, some theoretical constructs (eg, behavior change techniques [BCTs]) for changing participants’ sleep behaviors were applied. Also, some techniques derived from behavioral theories (eg, action planning and coping planning) were used to help participants achieve behavioral goals [47]. The app used in this study focuses on changing people’s sleep behaviors whereas other available sleep apps emphasize sleep mentoring [44]. Additionally, the apps summarized by Choi et al [44] generally assess sleep quantitatively (eg, sleep efficacy, sleep time) rather than taking sleep quality into account.

### Study Aims

This study aimed to determine the long-term treatment efficacy of a theory-based app using CBT, TPB, HAPA, and CT on sleep hygiene among insomnia patients. The primary evaluation involved comparing sleep hygiene behaviors and sleep quality with a control group that received patient education (PE).

## Methods

### Design

**Figure 1:**
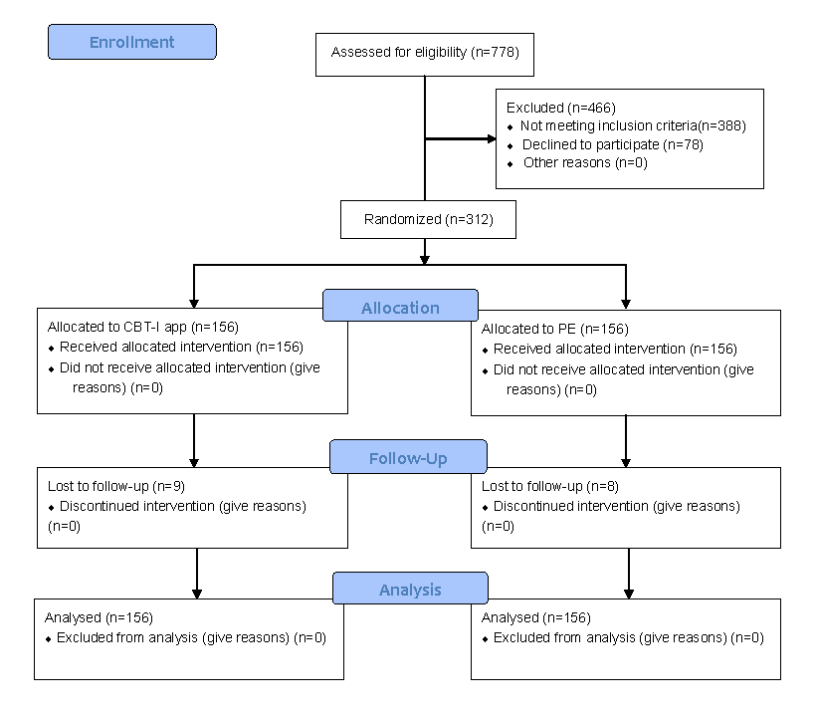
Flowchart of study design. CBT-I: cognitive behavioral therapy for insomnia; PE: patient education.

A 2-arm single-blind parallel-group RCT was launched to compare CBT-I and PE groups via an app over a 6-month period. Potential participants were included after initial screening and signing informed consent forms. The flowchart of the study design is shown in Figure 1. Primary and secondary outcomes were assessed online at baseline, 1 month postintervention, 3 months postintervention, and 6 months postintervention.

### Setting, Participants, and Recruitment

This Iranian-based study was advertised using brochures and posters at 3 universities, 5 colleges, and 10 general physicians’ offices as well as on social media. Interested participants were asked to access an online platform specifically designed for the study to complete a screening questionnaire assessing insomnia symptoms comprising the Insomnia Severity Index (ISI), sleep characteristics, and medical history as well as their time spent on computers and online. If participants met the initial criteria and expressed interest, a phone appointment was scheduled to conduct additional eligibility assessments. All telephone interviews were performed by three research assistants who had been trained by an experienced sleep medicine specialist during two 3-hour sessions. The sleep medicine specialist observed each research assistant during three actual patient encounters. The sleep medicine specialist and research assistant scored the patients independently. The scores were then compared to ensure that data collection procedures were consistent. There was more than 98% agreement between the sleep medicine specialist and research assistant in all cases.

### Selection Criteria

The inclusion criteria were (1) being age 18 years or older, (2) having insomnia disorder according to the *Diagnostic and Statistical Manual of Mental Disorders, Fifth Edition* (DSM-5) [48], (3) having an ISI score of 10 or higher [49], (4) understanding Persian, and (5) having access to a smartphone and/or desktop computer with internet access.

Participants were excluded if they (1) had an uncontrolled medical condition that interfered with sleep or required immediate treatment (eg, obstructive sleep apnea requiring continuous positive airway pressure treatment), (2) did shift work, (3) were pregnant, (4) were participating in other research and/or clinical trials, (5) had received psychotherapy in the past 6 months, (6) had current major depressive disorder based on the Structured Clinical Interview for DSM-5 disorders, (7) had a self-reported diagnosis of schizophrenia or psychosis, (8) showed evidence of alcohol abuse (more than 3 glasses of alcohol per day at least 21 days a month), (9) misused marijuana (use more than once per week), (10) appeared suicidal, or (11) had children aged under 2 years. After baseline assessments, participants were randomly assigned to a control group receiving PE or a treatment group receiving CBT-I. Both study groups had access to their related content on the app.

### Sample Size Calculation

The sample size was estimated based on previous studies on internet-based self-help insomnia interventions with a moderate effect size [42,50]. Using a 2-tailed test with a small-to-medium effect size (Cohen *d*=0.40) and significance level of *P*=.05, a total sample size of 266 (ie, 133 per group) had 90% power. To include an estimated dropout rate of 15%, entire sample size was increased from 266 to 312 participants (ie, 156 per group).

### Randomization and Allocation Procedures

Participants who met the inclusion criteria and signed the informed consent were randomly assigned to a control group (PE) or a treatment group (CBT-I) at a 1:1 ratio. Randomization was performed by an independent researcher via a random list generated using SPSS Statistics 24.0 (IBM Corp). Because blinding participants from treatment condition was impossible, the study blinded the data analyst using a code system for treatment condition. Moreover, the data analyst did not have access to the key document. Participants in both groups received assistance from a trained research assistant to help them install and unlock the app. To avoid contamination, participants in the PE group could not access the CBT-I content, which was locked using a personal code.

### App Overview

The app was designed based on a combination of TPB, HAPA, and CBT-I [29] and used the self-help concept [19] in order to treat insomnia. Several BCTs are integrated in the app, including information about health consequences, information about social and environmental consequences, habit formation, habit reversal, pros and cons of performing sleep hygiene behaviors, reconstructing the physical environment, reconstructing the social environment, self-monitoring of behavior, action planning, and problem solving [51]. The intervention contents were designed across 6 weeks, with exercises and subtutorials automatically provided each week (Table 1). Detailed information with screenshots is provided in Multimedia Appendix 1.

The content of the CBT-I was designed for treating patients with insomnia individually using weekly sessions, with each session lasting approximately 1 hour. However, participants could complete their sessions more quickly (ie, less than 1 hour) if they improved faster. The content of session 1 was provided for participants using plain text. For sessions 2 and 3, we used a variety of relaxation tools via audio guided meditation exercises as well as images. Session 4 provided correct information concerning sleep in plain text and tables, addressing incorrect information about insomnia participants may have heard. In sessions 5 and 6, plain text and tables were used to help participants plan their sleep hygiene behaviors. A sleep diary was used along with the ISI completed by participants to help them understand their daily sleep improvements. Development of the current app is still at an early stage, and customizing content to suit individual specific situations is under consideration for an updated version of the app. The current version does not have individually tailored content.

Access to the app content was provided to the participants each week; participants could not view the app content for weeks 2 through 6 (ie, sessions 2-6) when they were in week 1 of the intervention, but as participants continued, previous sessions remained unlocked. Every week, there was homework or an exercise, and participants were encouraged to complete it weekly. The content in the next session was opened the following week. Most of the participants used the app as recommended, and a reminder text message was sent to those who did not open the session content on time.

**Table 1:** Key intervention points for cognitive behavior therapy (BCT) used in the insomnia app.

Behavior change technique^a^	Targeted outcomes
BCT 5.3: information about social and environmental consequences (week 1)	Attitude and intentions to perform sleep hygiene behaviors
BCT 9.2: pros and cons (week 1)	Attitude and intentions to perform sleep hygiene behaviors
BCT 8.3: habit formation (week 2)	Behavioral automaticity
BCT 12.1: reconstructing the physical environment (week 2)	Perceived behavioral control
BCT 8.4: habit reversal (week 3)	Behavioral automaticity
BCT 12.3: reconstructing the social environment (week 3)	Perceived behavioral control
BCT 2.3: self-monitoring of behavior (week 4)	Self-monitoring
BCT 1.4: action planning (week 5)	Action planning
BCT 1.2: problem solving (week 5)	Coping planning

^a^Behavior change techniques are sourced from the taxonomy of Michie et al [51]. For instance, BCT 5.3 (information about social and environmental consequences) represents the subgroup of the taxonomy natural consequence (group 5).

### Patient Education Procedure

Participants in the PE group received written weekly information on accurate and relevant information regarding insomnia symptoms (week 1), physiological controls of sleep (week 2), sleep hygiene practices (week 3), healthy sleep behaviors (eg, reduce time in bed, get up at the same time every day, go to bed only if sleepy, and do not stay in bed unless asleep; week 4), and changing lifestyles to promote sleep health (week 5). The information was presented as separate content from the CBT-I in the app. This weekly information was unlocked for participants on a weekly basis. Participants in the PE group were informed that they could access the CBT-I content on the app at the end of the study (6 months after completing the intervention).

### Measures

All participants completed a sociodemographic profile questionnaire (age, gender, educational status, occupational status) at the baseline assessment and primary and secondary outcomes were assessed. The complete measures used to assess primary and secondary outcomes are shown in Multimedia Appendix 2.

#### Primary Outcomes

##### Sleep Hygiene Behavior

Sleep hygiene behavior was assessed using three items. The participants were asked to report how many days they had good sleep hygiene behavior over the past week (How many days did you make your bedroom restful over the past week?), whether they avoided going to bed feeling hungry or thirsty, and whether they avoided anxiety and stress-provoking activity before bed. Participants were asked to respond on an 8-point scale ranging from 0 to 7. The internal consistency of the three items was found to be acceptable in a previous study (α=.78) [38].

##### Insomnia Severity Index

The ISI is a 7-item self-report scale that assesses participant level of insomnia over the past 2 weeks. All items are rated on a 5-point Likert-type scale ranging from 0=no problem to 4=very severe problem. A total score is generated by summing all 7 items ranging from 0-28 with 5 subscores: 0-7 (absence of insomnia), 8-14 (subthreshold insomnia), 15-21 (moderate insomnia), and 22-28 (severe insomnia). The ISI has been translated into Persian, and its psychometric properties have been documented among Iranian adults [52].

##### Pittsburgh Sleep Quality Index

The Pittsburgh Sleep Quality Index (PSQI) is a subjective measure of sleep quality and disturbances over the past month that contains 19 items grouped into 7 separate component scores: subjective sleep quality, sleep latency, sleep duration, habitual sleep efficiency, sleep disturbances, use of sleeping medication, and daytime dysfunction. Scores are summed to provide a global sleep quality score. The Persian version of the PSQI has been validated and described in detail [53].

#### Secondary Outcomes

##### Attitude

Attitude toward good sleep hygiene was assessed using 5-point evaluative semantic differential scales (eg, “To make my bedroom/sleep environment restful would be: unpleasant- pleasant, good-bad, wise-foolish, correct-incorrect, unenjoyable- enjoyable, satisfying-unsatisfying, useful-useless”). Internal consistency of this 12-item scale has been found acceptable in previous studies [24,38].

##### Perceived Behavioral Control

Perceived behavioral control was assessed using three items (eg, “I am confident that every day I can prevent anxiety-provoking activity before bedtime”). All items were scored on a 5-point Likert scale, ranging from 1=totally disagree to 5=totally agree. The scale has been proved internally consistent in previous studies [24,38].

##### Behavioral Intention

Behavioral intention was assessed using 6 items (eg, “Over the next week, I intend to make my bedroom restful”). All items were scored on a 5-point Likert scale ranging from 1=totally disagree to 5=totally agree. Internal consistency of this scale has been found acceptable in previous studies [24,38].

##### Action Planning

Action planning was assessed using 4 items. The participants were asked to indicate if they have made a detailed plan regarding (1) when, (2) where, (3) how, and (4) how often they will perform sleep hygiene behaviors over the next 6 months. All items were scored on a 5-point Likert scale ranging from 1=totally disagree to 5=totally agree. The internal consistency of this scale has been found acceptable in previous studies [24,38,54].

##### Coping Planning

Coping planning was assessed using 5 items (eg, “I have made a detailed plan regarding what to do if something interferes with my plans”). All items were scored on a 5-point Likert scale ranging from 1=totally disagree to 5=totally agree. Internal consistency of this scale has been found acceptable in previous studies [24,38,55].

##### Self-Monitoring

Self-monitoring was assessed by 3 items (“I keep track of how much time I spend sleeping,” “I pay attention to how tired or rested I feel each day,” and “I take care to note the time that I go to bed and wake each day”). Responses were rated on a scale ranging from 1=never to 5=always.

##### Self-Report Behavioral Automaticity Index

The extent to which sleep hygiene behaviors are habitual for an individual was assessed using the Self-Report Behavioral Automaticity Index (SRBAI). The SRBAI contains 4 items that start with the stem “Sleep hygiene behavior is something...” followed by “I do automatically,” “I do without having to consciously remember,” “I do without thinking,” and “I start doing before I realize I’m doing it.” The reliability of the Persian SRBAI has been reported [56].

##### Hospital Anxiety and Depression Scale

The Hospital Anxiety and Depression Scale is a 14-item scale that assesses anxiety (7 items) and depression (7 items) in patients with both somatic and mental problems. All items were scored on a 0 to 3 scale with higher score representing higher levels of anxiety and depression. The Iranian version of the scale has been validated in different clinical patients [57].

### Data Management

All data were collected and stored using the FileMaker Pro 15 (Claris International Inc) database. Participants’ names, phone numbers, and addresses were recorded. Each participant received a unique code. All data were password protected and encrypted to ensure that confidentiality of the data was maintained throughout the study. The study was monitored (via quality control and audits) by the Social Determinants of Health Research Center at Qazvin University of Medical Sciences to ensure compliance with the protocol and applicable regulations was maintained and data were collected in a timely, accurate, and complete manner.

### Statistical Methods

All statistical analyses were performed in accordance with Consolidated Standards of Reporting Trials (CONSORT) guidelines. Intention-to-treat analyses were performed to take care of attrition. Descriptive statistics were used to summarize the characteristics of the participants. To evaluate the magnitude of changes in primary and secondary outcomes over time across the two groups, linear mixed models (PROC MIXED) were performed controlling for baseline variables and other covariates that may relate to the outcome. The mixed modeling approach is a powerful statistical tool to evaluate group differences over time with unequal numbers of participants at baseline and follow-up. It is also a helpful way to handle missing data using full information maximum likelihood estimation. The analysis incorporated two between-participant effects (between groups and between participants within groups) and three within-participant effects (between times, group by time interactions, and random variation). All *P* values were 2-sided and were evaluated as statistically significant at the .05 level. All statistical analyses were performed using SAS version 9.3 (SAS Institute Inc).

### Ethics and Dissemination

This study protocol was approved by the ethics committee of Qazvin University of Medical Sciences (IR.QUMS.REC. 1396.455) and is registered with ClinicalTrials.gov [NCT03605732], last updated July 2018. This study was performed based on the Helsinki Declaration principles. Required permissions were obtained from authorities of Qazvin University of Medical Sciences. After research assistants expressed objectives and assured participants about the confidentiality of their data and possibility of withdrawing from the study, informed consent forms were signed by those participants willing to participate in this research.

## Results

### General Use of the App

App engagement was assessed using an online database that recorded the number of log-ins and the duration of each log-in for all participants. The results showed that over the 6-month study period, the mean number of log-ins in both groups was 41.21 (SD 39.50). Participants in the CBT group had a higher number of log-ins (mean 42.65 [SD 42.58]) as compared with the PE group (mean 40.93 [SD 45.01]); however, this difference was not statistically significant (*P*=.38). Mean length of time using the app in all participants was 1580 minutes (SD 1460.37). The participants in the CBT group had significantly longer duration of app use (mean 1780.34 [SD 1770.07] minutes) compared with the PE group (mean 1370 [SD 450.01] minutes; *P*=.03).

### Descriptive Statistics in Demographics, Primary Outcomes, and Secondary Outcomes

Table 2 shows that both CBT-I and PE groups shared similar demographic characteristics. Specifically, the mean age of the insomnia patients in the CBT-I group was 36.21 (SD 5.81) years, and the mean age of those in the PE was 35.29 (SD 5.76) years. Slightly less than half of the participants were male in each group (46.1% [72/156] in the CBT-I group and 42.3% [66/156] in the PE group).

Table 3 describes the sleep quality (using PSQI), severity of insomnia (using ISI), sleep hygiene behaviors, and health status of both groups at baseline and 1-month, 3-month, and 6-month follow-ups.

**Table 2:** Demographic characteristics by condition (n=312).

Characteristic	PE^a^	CBT-I^b^	*P* value
Age in years, mean (SD)	35.29 (5.76)	36.21 (5.81)	.16
Male, n (%)	66 (42.3)	72 (46.1)	.49
Education in years, mean (SD)	14.22 (4.28)	14.70 (5.13)	.37
Insomnia duration in months, mean (SD)	16.71 (5.33)	15.89 (7.02)	.25

^a^PE: patient education.

^b^CBT-I: cognitive behavioral therapy for insomnia.

**Table 3:** Descriptive statistics for all outcome measures by condition and time.

Outcome	PE^a^ mean (SD)				CBT-I^b^ mean (SD)				
	Baseline	Month 1	Month 3	Month 6		Baseline	Month 1	Month 3	Month 6
PSQI^c^	13.34 (2.72)	12.23 (3.79)	12.42 (3.88)	12.34 (3.92)		13.25 (3.13)	9.35 (4.82)	9.33 (3.82)	9.09 (4.02)
ISI^d^	19.21 (4.57)	16.69 (4.98)	16.65 (4.77)	16.70 (5.63)		19.26 (4.57)	12.67 (5.60)	12.50 (5.57)	12.38 (5.55)
Sleep hygiene behavior	9.27 (2.46)	12.84 (5.74)	12.90 (5.27)	13.00 (4.25)		9.86 (3.14)	15.61 (5.95)	15.57 (5.12)	15.82 (5.10)
Attitude	2.69 (0.77)	3.22 (0.97)	3.20 (1.02)	3.23 (1.19)		2.74 (0.59)	3.61 (0.76)	3.66 (0.63)	3.68 (0.72)
PBC^e^	2.81 (0.94)	2.97 (0.74)	2.94 (1.01)	2.88 (0.68)		2.77 (0.86)	4.20 (0.84)	4.17 (0.59)	4.24 (0.89)
Intention	2.82 (1.15)	3.08 (1.08)	3.12 (1.14)	3.14 (1.11)		2.87 (1.02)	4.33 (0.71)	4.37 (1.06)	4.40 (1.09)
Action planning	2.12 (0.63)	2.36 (1.14)	2.38 (1.18)	2.34 (1.22)		2.18 (0.87)	3.56 (1.11)	3.67 (0.91)	3.64 (1.02)
Coping planning	2.48 (0.68)	2.83 (1.16)	2.86 (1.14)	2.89 (1.21)		2.43 (0.98)	3.75 (0.81)	3.92 (0.71)	3.96 (0.77)
Self-monitoring	2.29 (0.90)	2.84 (0.97)	2.85 (0.99)	2.88 (0.91)		2.32 (0.94)	3.32 (1.04)	3.41 (1.11)	3.43 (1.16)
SRBAI^f^	3.37 (1.20)	3.05 (1.16)	3.24 (1.06)	3.20 (1.24)		3.23 (1.12)	4.50 (0.89)	4.48 (0.18)	4.52 (0.79)
Anxiety^g^	8.35 (3.62)	7.82 (4.13)	7.93 (4.91)	7.96 (4.88)		8.22 (3.89)	5.58 (3.84)	5.27 (3.63)	5.50 (3.69)
Depression^g^	6.03 (3.30)	5.98 (3.13)	5.94 (3.28)	5.97 (3.25)		6.30 (3.82)	3.81 (0.89)	3.36 (0.94)	3.22 (0.88)

^a^PE: patient education.

^b^CBT-I: cognitive behavioral therapy for insomnia.

^c^PSQI: Pittsburgh Sleep Quality Index.

^d^ISI: Insomnia Severity Index.

^e^PBC: perceived behavioral control.

^f^SRBAI: Self-Report Behavioral Automaticity Index.

^g^Anxiety and depression were measured using the Hospital Anxiety and Depression Scale.

### Effects of the Intervention on Primary Outcomes

The intervention had promising effects on the three primary outcomes: sleep quality, severity of insomnia, and sleep hygiene behaviors. Specifically, sleep quality was improved among insomnia patients in the CBT-I group compared with those in the PE at all follow-ups, as suggested by scores on the PSQI (*P*<.001 for 1, 3, and 6 months). Similar improvements were shown in severity of insomnia (ie, significant decrease in the severity of insomnia among the CBT-I group compared with those in the PE group at 1, 3, and 6 months following the intervention, all *P* values <.001) and sleep hygiene behaviors (insomnia patients in the CBT-I group significantly engaged in more good sleep hygiene behaviors than those in the PE group at 1 [*P*=.02], 3 [*P*=.04], and 6 [*P*=.02] months following the intervention). Table 4 shows the findings of the linear mixed models predicting primary outcomes between two groups after controlling for their age, gender, and education.

**Table 4:** Results of the linear mixed models for primary outcomes.

Variables	PSQI^a^		ISI^b^		Sleep hygiene behaviors		
	B^c^ (SE)	*P* value (95% CI)	B (SE)	*P* value (95% CI)		B (SE)	*P* value (95% CI)
CBT-I^d^ (vs PE^e^ at baseline)	–0.02 (0.55)	.97 (–1.10 to 1.06)	0.05 (0.51)	.92 (–0.95 to 1.05)		0.40 (0.66)	.55 (–0.89 to 1.69)
Month 1 (vs baseline)	–1.11 (0.46)	.02 (–2.01 to –0.21)	–2.52 (0.37)	<.001 (–3.25 to –1.80)		3.56 (0.62)	<.001 (2.35 to 4.78)
Month 3 (vs baseline)	–0.92 (0.42)	.03 (–1.74 to –0.10)	–2.55 (0.36)	<.001 (–3.26 to –1.84)		3.64 (0.63)	<.001 (2.41 to 4.88)
Month 6 (vs baseline)	–1.0 (0.39)	.01 (–1.76 to –0.24)	–2.51 (0.44)	<.001 (–3.37 to –1.65)		3.73 (0.69)	<.001 (2.38 to 5.08)
CBT-I (vs PE at 1 month)	–2.76 (0.64)	<.001 (–4.01 to –1.51)	–4.01 (0.51)	<.001 (–5.01 to –3.01)		2.19 (0.93)	.02 (0.37 to 4.01)
CBT-I (vs PE at 3 months)	–3.0 (0.66)	<.001 (–4.29 to –1.71)	–4.21 (0.56)	<.001 (–5.31 to –3.11)		2.01 (0.96)	.04 (0.13 to 3.89)
CBT-I (vs PE at 6 months)	–3.16 (0.61)	<.001 (–4.36 to –1.96)	–4.36 (0.50)	<.001 (–5.34 to –3.38)		2.23 (0.91)	.02 (0.45 to 4.01)
Age	0.03 (0.03)	.32 (–0.03 to 0.09)	0.02 (0.04)	.62 (–0.06 to 0.10)		0.09 (0.04)	.03 (0.01 to 0.17)
Female	0.31 (0.40)	.44 (–0.47 to 1.09)	0.04 (0.42)	.92 (–0.78 to 0.86)		0.13 (0.35)	.71 (–0.56 to 0.82)
Education	0.44 (0.72)	.54 (–0.97 to 1.85)	0.22 (0.38)	.56 (–0.53 to 0.97)		0.70 (0.54)	.20 (–0.36 to 1.76)
Intercept	14.14 (1.23)	<.001 (11.73 to 16.55)	18.67 (1.27)	<.001 (16.18 to 21.16)		5.99 (1.13)	<.001 (3.78 to 8.21)

^a^PSQI: Pittsburgh Sleep Quality Index.

^b^ISI: Insomnia Severity Index.

^c^B: unstandardized coefficient.

^d^CBT-I: cognitive behavioral therapy for insomnia.

^e^PE: patient education.

### Effects of the Intervention on Secondary Outcomes

In terms of secondary outcomes, analyses indicated that insomnia patients in the CBT-I group had better attitude (*P*=.03 at 1 month; *P*=.04 at 3 months; *P*=.03 at 6 months), stronger perceived behavioral control (*P* values <.001 at 1, 3, and 6 months), higher intention (*P* values <.001 at 1, 3, and 6 months) to develop good sleep hygiene behavior, and were more likely to have formed relevant action and coping plans (*P* values <.001 at 1, 3, and 6 months; Table 5). Additionally, insomnia patients in the CBT-I group had higher behavioral automaticity (*P* values <.001 at 1, 3, and 6 months), better self-monitoring (*P* values ≤.001 at 1, 3, and 6 months), and less anxiety (*P*=.003 at 1 month and *P*<.001 at both 3 and 6 months), and depression (*P* values <.001 at 1, 3, and 6 months) relative to those in the PE group (Table 6).

**Table 5:** Results of the linear mixed models for measures on theory of planned behavior and health action process approach concepts.

Variables	Attitude		PBC^a^		Intention		Action planning			Coping planning	
	B^b^ (SE)	*P* value (95% CI)	B (SE)	*P* value (95% CI)	B (SE)	*P* value (95% CI)	B (SE)	*P* value (95% CI)	B (SE)		*P* value (95% CI)
CBT-I^c^ (vs PE^d^)	0.02 (0.13)	.88 (–0.24 to 0.28)	0.05 (0.14)	.72 (–0.22 to 0.32)	0.03 (0.16)	.85 (–0.28 to 0.34)	0.04 (0.16)	.80 (–0.27 to 0.35)	0.04 (0.15)		.79 (–0.25 to 0.33)
Month 1 (vs baseline)	0.53 (0.11)	<.001 (0.31 to 0.75)	0.16 (0.12)	.18 (–0.08 to 0.40)	0.25 (0.14)	.08 (–0.02 to 0.52)	0.24 (0.12)	.046 (0.005 to 0.48)	0.36 (0.13)		.01 (0.11 to 0.62)
Month 3 (vs baseline)	0.51 (0.16)	.002 (0.20 to 0.82)	0.15 (0.16)	.35 (–0.16 to 0.46)	0.29 (0.15)	.05 (–0.004 to 0.58)	0.26 (0.14)	.06 (–0.01 to 0.53)	0.39 (0.12)		.001 (0.16 to 0.63)
Month 6 (vs baseline)	0.53 (0.18)	.003 (0.18 to 0.88)	0.08 (0.11)	.47 (–0.14 to 0.30)	0.31 (0.18)	.09 (–0.04 to 0.66)	0.22 (0.11)	.046 (0.004 to 0.44)	0.42 (0.14)		.003 (0.15 to 0.69)
CBT-I (vs PE at 1 month)	0.34 (0.16)	.03 (0.03 to 0.65)	1.27 (0.17)	<.001 (0.94 to 1.60)	1.21 (0.20)	<.001 (0.82 to 1.60)	1.13 (0.17)	<.001 (0.80 to 1.46)	0.91 (0.17)		<.001 (0.58 to 1.24)
CBT-I (vs PE at 3 months)	0.40 (0.19)	.04 (0.03 to 0.77)	1.25 (0.15)	<.001 (0.96 to 1.54)	1.11 (0.22)	<.001 (0.68 to 1.54)	1.22 (0.13)	<.001 (0.97 to 1.48)	1.06 (0.16)		<.001 (0.75 to 1.37)
CBT-I (vs PE at 6 months)	0.39 (0.18)	.03 (0.04 to 0.74)	1.40 (0.14)	<.001 (1.13 to 1.67)	1.02 (0.21)	<.001 (0.61 to 1.43)	1.26 (0.19)	<.001 (0.89 to 1.63)	1.10 (0.15)		<.001 (0.81 to 1.39)
Age	–0.01 (0.01)	.32 (–0.03 to 0.01)	0.03 (0.07)	.67 (–0.11 to 0.17)	–0.05 (0.09)	.58 (–0.23 to 0.13)	0.01 (0.05)	.84 (–0.09 to 0.11)	0.04 (0.09)		.66 (–0.14 to 0.22)
Female	–0.08 (0.09)	.38 (–0.26 to 0.10)	0.16 (0.09)	.08 (–0.02 to 0.34)	0.14 (0.11)	.20 (–0.08 to 0.36)	0.11 (0.12)	.36 (–0.13 to 0.35)	0.09 (0.11)		.41 (–0.13 to 0.31)
Education	0.27 (0.14)	.06 (–0.004 to 0.54)	0.06 (0.08)	.45 (–0.10 to 0.22)	0.28 (0.18)	.12 (–0.07 to 0.63)	0.27 (0.21)	.20 (–0.14 to 0.68)	0.05 (0.18)		.78 (–0.30 to 0.40)
Intercept	2.53 (0.26)	<.001 (2.02 to 3.04)	2.66 (0.28)	<.001 (2.11 to 3.21)	2.82 (0.35)	<.001 (2.13 to 3.51)	1.76 (0.40)	<.001 (0.98 to 2.54)	2.46 (0.34)		<.001 (1.79 to 3.13)

^a^PBC: perceived behavioral control.

^b^B: unstandardized coefficient.

^c^CBT-I: cognitive behavioral therapy for insomnia.

^d^PE: patient education.

**Table 6:** Results of the linear mixed models for measures on control theory concept and psychological distress (anxiety and depression).

Variables	SRBAI^a^		Self-monitoring		Anxiety		Depression	
	B^b^ (SE)	*P* value (95% CI)	B (SE)	*P* value (95% CI)	B (SE)	*P* value (95% CI)	B (SE)	*P* value (95% CI)
CBT-I^c^ (vs PE^d^)	0.12 (0.19)	.53 (–0.25 to 0.49)	0.03 (0.14)	.83 (–0.24 to 0.30)	–0.02 (0.56)	.97 (–1.12 to 1.08)	–0.27 (0.47)	.57 (–1.19 to 0.65)
Month 1 (vs baseline)	0.32 (0.16)	.046 (0.01 to 0.63)	0.56 (0.09)	<.001 (0.38 to 0.74)	–0.53 (0.51)	.30 (–1.53 to 0.47)	–0.04 (0.44)	.93 (–0.90 to 0.82)
Month 3 (vs baseline)	0.43 (0.18)	.02 (0.08 to 0.78)	0.57 (0.11)	<.001 (0.35 to 0.79)	–0.41 (0.50)	.41 (–1.39 to 0.57)	–0.08 (0.37)	.83 (–0.81 to 0.65)
Month 6 (vs baseline)	0.47 (0.21)	.03 (0.06 to 0.88)	0.58 (0.10)	<.001 (0.38 to 0.78)	–0.38 (0.46)	.41 (–1.28 to 0.52)	–0.05 (0.35)	.89 (–0.74 to 0.64)
CBT-I (vs PE at 1 month)	1.59 (0.23)	<.001 (1.14 to 2.04)	0.43 (0.13)	.001 (0.18 to 0.69)	–2.12 (0.72)	.003 (–3.53 to –0.71)	–2.44 (0.62)	<.001 (–3.66 to –1.23)
CBT-I (vs PE at 3 months)	1.68 (0.22)	<.001 (1.25 to 2.11)	0.52 (0.12)	<.001 (0.29 to 0.76)	–2.53 (0.70)	<.001 (–3.90 to –1.16)	–2.86 (0.61)	<.001 (–4.06 to –1.66)
CBT-I (vs PE at 6 months)	1.76 (0.26)	<.001 (1.25 to 2.27)	0.54 (0.16)	.001 (0.23 to 0.85)	–2.64 (0.75)	<.001 (–4.11 to –1.17)	–3.03 (0.65)	<.001 (–4.30 to –1.76)
Age	–0.01 (0.01)	.32 (–0.03 to 0.01)	–0.06 (0.10)	.55 (–0.26 to 0.14)	–0.05 (0.03)	.10 (–0.11 to 0.01)	–0.01 (0.02)	.62 (–0.05 to 0.03)
Female	0.14 (0.13)	.28 (–0.12 to 0.40)	0.18 (0.12)	.14 (–0.06 to 0.42)	0.03 (0.27)	.91 (–0.50 to 0.56)	0.26 (0.30)	.39 (–0.33 to 0.85)
Education	0.06 (0.10)	.55 (–0.14 to 0.26)	0.53 (0.26)	.042 (0.02 to 1.04)	0.53 (0.61)	.39 (–0.67 to 1.73)	0.54 (0.45)	.23 (–0.34 to 1.42)
Intercept	3.81 (0.41)	<.001 (3.01 to 4.61)	2.23 (0.37)	<.001 (1.51 to 2.96)	9.86 (1.18)	<.001 (7.55 to 12.17)	5.92 (0.94)	<.001 (4.08 to 7.76)

^a^SRBAI: Self-Reported Behavioral Automaticity Index.

^b^B: unstandardized coefficient.

^c^CBT-I: cognitive behavioral therapy for insomnia.

^d^PE: patient education.

Mediation analyses were conducted to investigate whether the effects of CBT-I on the primary outcome of sleep hygiene behaviors were mediated by changes in relevant beliefs about sleep hygiene behaviors as specified by the three proposed theories (TPB, HAPA, and CT). Regarding sleep hygiene behaviors at 3 months, only action planning was a significant mediator (coefficient 1.11; 95% CI 0.54-1.69). Regarding sleep hygiene behaviors at 6 months, perceived behavioral control (coefficient 0.44; 95% CI 0.10-0.78), coping planning (coefficient 0.24; 95% CI 0.12-0.36), self-monitoring (coefficient 0.58; 95% CI 0.13-1.03), and behavioral automaticity (coefficient 1.00; 95% CI 0.17-1.83) were significant mediators (Table 7).

**Table 7:** Mediated effects of variables in theory of planned behavior, health action process approach, and control theory in the impacts of the cognitive behavioral therapy for insomnia app intervention on self-reported sleep hygiene behaviors at 3 months postintervention.

Outcome and mediator		Intervention effect on outcome (C) (SE/95% CI)	Intervention effect on mediator (A) (SE/95% CI)	Mediator effect on outcome (B) (SE/95% CI)	Mediated effect (A^*^B) (SE/95% CI)
**At 3 months**					
	Sleep hygiene behaviors	2.01 (0.96/0.12 to 3.09)			
	Attitude		0.34 (0.16/0.02 to 0.66)	1.71 (0.65/0.43 to 2.99)	0.58 (0.35/–0.11 to 1.27)
	PBC^a^		1.27 (0.17/1.07 to 1.47)	1.67 (1.11/–0.52 to 3.86)	2.12 (1.44/–0.72 to 4.96)
	Intention		1.21 (0.20/0.82 to 1.60)	0.24 (0.67/–1.08 to 1.56)	0.29 (0.81/–1.31 to 1.89)
	Action planning		1.13 (0.17/0.79 to 1.47)	0.98 (0.21/0.57 to 1.39)	1.11 (0.29/0.54 to 1.69)
	Coping planning		0.91 (0.17/0.57 to 1.25)	0.35 (0.62/–0.87 to 1.57)	0.32 (0.57/–0.80 to 1.45)
	Self-monitoring		0.43 (0.13/0.17 to 0.69)	1.79 (0.48/0.84 to 2.74)	0.77 (0.42/–0.06 to 1.60)
	SRBAI^b^		1.59 (0.23/1.14 to 2.04)	0.10 (0.25/–0.39 to 0.59)	0.16 (0.40/–0.63 to 0.95)
**At 6 months**					
	Sleep hygiene behaviors	2.23 (0.91/0.43 to 4.02)			
	Attitude		0.34 (0.16/0.02 to 0.66)	0.93 (0.37/0.21 to 1.66)	0.32 (0.19/–0.05 to 0.69)
	PBC		1.27 (0.17/1.07 to 1.47)	0.34 (0.13/0.08 to 0.60)	0.44 (0.17/0.10 to 0.78)
	Intention		1.21 (0.20/0.82 to 1.60)	0.07 (0.33/0.58 to 0.71)	0.08 (0.40/–0.71 to 0.87)
	Action planning		1.13 (0.17/0.79 to 1.47)	0.25 (0.24/0.22 to 0.73)	0.28 (0.27/–0.25 to 0.81)
	Coping planning		0.91 (0.17/0.57 to 1.25)	0.26 (0.05/0.16 to 0.36)	0.24 (0.06/0.12 to 0.36)
	Self-monitoring		0.43 (0.13/0.17 to 0.69)	1.36 (0.33/0.71 to 2.01)	0.58 (0.23/0.13 to 1.03)
	SRBAI		1.59 (0.23/1.14 to 2.04)	0.63 (0.25/0.14 to 1.12)	1.00 (0.42/0.17 to 1.83)

^a^PBC: perceived behavioral control.

^b^SRBAI: Self-Reported Behavioral Automaticity Index.

## Discussion

### Principal Findings

This research found that a theory-based intervention improved sleep outcomes among insomnia patients in Iran, as evidenced by improved sleep hygiene behaviors, increased sleep quality, and decreased insomnia severity. These beneficial effects were mediated by several changes in the putative determinants of behavior, including perceived behavioral control in TPB, action and coping planning in HAPA, and self-monitoring and behavioral automaticity in CT. Moreover, the entire treatment effects were guided by CBT (ie, the use of BCTs). Furthermore, as a consequence of improvements in sleep, positive effects of CBT-I were demonstrated by reduced anxiety and depression among insomnia patients. Researchers and practitioners interested in improving sleep quality, particularly among insomnia patients, might draw on insights provided by several components in TPB [7,35-38], HAPA [38,39], and CT [40] in order to design effective interventions for insomnia patients. Moreover, use of the CBT-I app may enhance the feasibility of providing CBT-I treatments incorporating the components in TPB, HAPA, and CT mentioned above.

The finding that the PE group did not have substantial improvement could be due to the following barriers. The written information (1) did not have interactive components, commonly used in apps, and may not have triggered the motivation of participants to obtain the information; (2) did not have any psychological mechanism to support its use whereas the CBT-I app incorporated and improved psychological components (eg, attitudes) for participants to actively engage in sleep, and (3) may have been hard to translate into daily practice for the PE group. Consequently, use of the PE group as a control is appropriate because PE only provides passive engagement and does not have any psychological mechanism to support its use. On the other hand, the CBT-I app involved active engagement and included psychological mechanism to support its use.

Apart from illustrating the effectiveness of the CBT-I app, our findings support the notion that the improvements in sleep accrued (in part) from changes in several components specified by TPB (perceived behavioral control), HAPA (action and coping planning), and CT (self-monitoring and behavioral automaticity). Therefore, our findings partially support the notions from previous studies: health care providers may want to use TPB to effectively understand the performance of sleep hygiene behaviors [37,38]. Additionally, these findings go beyond those correlational studies [37,38] and agree with another RCT study [24] that used theory-based (TPB, HAPA, and CBT) interventions to improve sleep hygiene behaviors among adolescents. More specifically, changes in the putative determinants of action lead to changes in the respective behaviors (eg, sleep hygiene behaviors, sleep quality, and severity of insomnia). Consequently, this research provides experimental support for the combination of TPB, HAPA, CT, and CBT as a framework for improving sleep among insomnia patients.

Our results also support the idea that it is important for CBT-I interventions to incorporate self-regulatory processes such as those specified in HAPA and CT. More specifically, action planning, coping planning, self-monitoring, and behavioral automaticity were significant mediators in this study. Interestingly, mediated effects of action planning on sleep hygiene behaviors were found at 3 months after completing the CBT-I program but not at 6 months after completing the program. In contrast, mediated effects of coping planning, self-monitoring, and behavioral automaticity on sleep hygiene behaviors were observed at 6 months after completing the CBT-I program but not at 3 months after completing the program.

A possible explanation is the cognitive progress during the CBT-I program. Insomnia patients first need to generate effective strategies (ie, using action planning) to assist them in developing good sleep hygiene behaviors because they do not have sufficient ability to devise good strategies [24]. Therefore, action planning was an important mediator for sleep hygiene behaviors at 3 months. Meanwhile, coping planning, self-monitoring, and behavioral automaticity are not mediators at this stage because insomnia patients put all their efforts in action planning and do not have additional efforts to work on coping planning, self-monitoring, and behavioral automaticity. In terms of sleep hygiene behaviors at 6 months, insomnia patients are very likely to have satisfactory capability in action planning. Therefore, action planning loses its importance as mediator because insomnia patients can use the good strategies they have generated. In contrast, insomnia patients need to deal with all the unforeseen barriers and keep their practice. Therefore, coping planning (ie, thinking ahead to tackle possible barriers) [39], self-monitoring (ie, reflection on the difference between preferred and actual performance) [40], and behavioral automaticity (ie, developing the behaviors into habitual pattern) [56] become important mediators at this stage.

This study’s findings on self-regulatory processes also support those of correlational studies that point to the importance of self-regulatory processes in predicting sleep hygiene behaviors and related outcomes, although in a different population [38]. In their study with obstructive sleep apnea patients, Deng et al [58] found that an intervention based on HAPA improved participant sleep quality. Our study suggests that targeting different cognitive processes (ie, motivational and self-regulatory processes) specified in TPB, HAPA, and HAPA can lead to changes in the respective behaviors (eg, sleep hygiene in this study).

### Strengths and Limitations

Our study has several strengths. First, few trials using psychological interventions built on empirically based theories have been designed for sleep problems. Therefore, results derived from this study provided information about relevant mechanisms of change. Second, development of an app may improve the effects of a sleep intervention in insomnia. More specifically, providing a technology-based intervention could be an effective and accessible way to overcome numerous barriers to CBT-I, such as low access, high costs, time constraints, social stigma, and the lack of therapists and trained mental health experts, as well as providing an intervention overcoming economic and geographical barriers. Third, this study controlled for many potential biases. For example, the blindness of the analyst and the avoidance of contamination were addressed. Fourth, although all the measures were self-reported, instruments used in this study had satisfactory psychometric properties. Consequently, the reliability and validity of outcome measures were ensured.

There are some limitations in the study. First, participants were not representative of the entire population of Iranian patients with insomnia. For example, participants in this study needed to use the app to receive the intervention. Therefore, insomnia patients who have little literacy in using smartphones or desktop computers may not gain benefit from CBT-I. Given that the mean age of the studied sample was mid-thirties; the possibility to generalize our findings to all inhabitants and age groups is low. Second, the use of self-report measures for sleep and mental health outcomes could be biased by social desirability or memory recall. Although mental health outcomes are hard to measure using non-self-reports, future studies may want to use objectively measured instruments on sleep quality (eg, actigraphy). Third, participants could not be blinded because the intervention was obvious to them. Therefore, placebo effects were hard to exclude, especially because most of the outcome measures were self-reported. However, given that the promising effects were found across all outcome measures and placebo effects would be unlikely to last 6 months after treatment ends, we tentatively concluded that CBT-I is an effective program to treat insomnia. Last, as the study only followed participants 6 months after completing the intervention, it is unclear whether CBT-I can provide effects lasting longer than 6 months. Future studies are thus warranted to examine the long-term effect of CBT-I.

### Conclusion

The theory-based CBT-I intervention shows promising effects in treating sleep problems for insomnia patients. After receiving the feasible and short (ie, 6 weeks) CBT-I, insomnia patients showed improved sleep hygiene behaviors, enhanced sleep quality, and less insomnia severity. Moreover, other psychological distress outcomes (ie, anxiety and depression) of the insomnia patients who received CBT-I showed improvement lasting 6 months after the intervention ended.

## Appendix

Multimedia Appendix 1 Behavior change techniques, content, and screenshots of app.

Multimedia Appendix 2 Questionnaires used to assess primary and secondary outcomes.

Multimedia Appendix 3 CONSORT-eHEALTH checklist (V 1.6.1).

## Abbreviations

BCT: behavior change technique
CBT: cognitive behavioral therapy
CBT-I: cognitive behavioral therapy for insomnia
CONSORT: Consolidated Standards of Reporting Trials
CT: control theory
DSM-5: Diagnostic and Statistical Manual of Mental Disorders, Fifth Edition
HAPA: health action process approach
ISI: Insomnia Severity Index
PE: patient education
PSQI: Pittsburgh Sleep Quality Index
RCT: randomized controlled trial
SRBAI: Self-Report Behavioral Automaticity Index
TPB: theory of planned behavior

## References

[1] Barati Dowom P, Roshanaei K, Darvishi M (2015). Neurophysiological mechanism of sleep and wakefulness regulation. Neurosci J Shefaye Khatam.

[2] Septianto R (2014). Relationship Between Depression Level With Score of Clinical Reasoning Module I in the Medical Students UIN Syarif Hidayatullah Jakarta Class 2013 [Thesis].

[3] Ohayon MM, Roth T (2003). Place of chronic insomnia in the course of depressive and anxiety disorders. J Psychiatr Res.

[4] Simon GE, VonKorff M (1997). Prevalence, burden, and treatment of insomnia in primary care. Am J Psychiatry.

[5] Babamiri M, Moeini B, Tahmasian H, Barati M, Roshanai G (2017). The study of sleep health education effect on sleep quality among Lorestan nursing personnel. J Ergon.

[6] National Sleep Foundation.

[7] Kor K, Mullan BA (2011). Sleep hygiene behaviours: an application of the theory of planned behaviour and the investigation of perceived autonomy support, past behaviour and response inhibition. Psychol Health.

[8] Williams R (2017). The Prevalence of Insomnia on School Principals and Superintendents in Missouri [Thesis].

[9] Bjorvatn B, Mrdalj J, Saxvig IW, Aasnæs T, Pallesen S, Waage S (2017). Age and sex differences in bedroom habits and bedroom preferences. Sleep Med.

[10] Araújo T, Jarrin DC, Leanza Y, Vallières A, Morin CM (2017). Qualitative studies of insomnia: current state of knowledge in the field. Sleep Med Rev.

[11] Webb TL, Joseph J, Yardley L, Michie S (2010). Using the internet to promote health behavior change: a systematic review and meta-analysis of the impact of theoretical basis, use of behavior change techniques, and mode of delivery on efficacy. J Med Internet Res.

[12] Morin CM, Beaulieu-Bonneau S, LeBlanc M, Savard J (2005). Self-help treatment for insomnia: a randomized controlled trial. Sleep.

[13] Bonnar D, Gradisar M, Moseley L, Coughlin A, Cain N, Short MA (2015). Evaluation of novel school-based interventions for adolescent sleep problems: does parental involvement and bright light improve outcomes?. Sleep Health.

[14] Cain N, Gradisar M, Moseley L (2011). A motivational school-based intervention for adolescent sleep problems. Sleep Med.

[15] Gooneratne N (2007). Complementary and Alternative Medicine for Sleep Disturbances [Thesis].

[16] Roehrs T, Roth T (2001). Sleep, sleepiness, and alcohol use. Alcohol Res Health.

[17] van Straten A, Cuijpers P (2009). Self-help therapy for insomnia: a meta-analysis. Sleep Med Rev.

[18] Cushing CC, Jensen CD, Miller MB, Leffingwell TR (2014). Meta-analysis of motivational interviewing for adolescent health behavior: efficacy beyond substance use. J Consult Clin Psychol.

[19] Gruber R, Somerville G, Bergmame L, Fontil L, Paquin S (2016). School-based sleep education program improves sleep and academic performance of school-age children. Sleep Med.

[20] Rigney G, Blunden S, Maher C, Dollman J, Parvazian S, Matricciani L, Olds T (2015). Can a school-based sleep education programme improve sleep knowledge, hygiene and behaviours using a randomised controlled trial. Sleep Med.

[21] Prestwich A, Sniehotta FF, Whittington C, Dombrowski SU, Rogers L, Michie S (2014). Does theory influence the effectiveness of health behavior interventions? Meta-analysis. Health Psychol.

[22] Prestwich A, Webb TL, Conner M (2015). Using theory to develop and test interventions to promote changes in health behaviour: evidence, issues, and recommendations. Curr Opin Psychol.

[23] Rothman AJ (2004). “Is there nothing more practical than a good theory?” Why innovations and advances in health behavior change will arise if interventions are used to test and refine theory. Int J Behav Nutr Phys Act.

[24] Lin C, Strong C, Scott A, Broström A, Pakpour A, Webb T (2018). A cluster randomized controlled trial of a theory-based sleep hygiene intervention for adolescents. Sleep.

[25] Drake CL (2016). The promise of digital CBT-I. Sleep.

[26] Manber R, Carney C (2015). Treatment Plans and Interventions for Insomnia: A Case Formulation Approach.

[27] Merrigan JM, Buysse DJ, Bird JC, Livingston EH (2013). Insomnia. JAMA.

[28] Morin CM, Vallières A, Guay B, Ivers H, Savard J, Mérette C, Bastien C, Baillargeon L (2009). Cognitive behavioral therapy, singly and combined with medication, for persistent insomnia: a randomized controlled trial. JAMA.

[29] Richardson R, Richards DA (2005). Self help: towards the next generation. Behav Cognit Psychother.

[30] Jernelöv S, Lekander M, Blom K, Rydh S, Ljótsson B, Axelsson J, Kaldo V (2012). Efficacy of a behavioral self-help treatment with or without therapist guidance for co-morbid and primary insomnia: a randomized controlled trial. BMC Psychiatry.

[31] Insel TR, Cuthbert BN (2009). Endophenotypes: bridging genomic complexity and disorder heterogeneity. Biol Psychiatry.

[32] Leichsenring F, Hiller W, Weissberg M, Leibing E (2006). Cognitive-behavioral therapy and psychodynamic psychotherapy: techniques, efficacy, and indications. Am J Psychother.

[33] Seyffert M, Lagisetty P, Landgraf J, Chopra V, Pfeiffer PN, Conte ML, Rogers MAM (2016). Internet-delivered cognitive behavioral therapy to treat insomnia: a systematic review and meta-analysis. PLoS One.

[34] Ye Y, Zhang Y, Chen J, Liu J, Li X, Liu Y, Lang Y, Lin L, Yang X, Jiang X (2015). Internet-based cognitive behavioral therapy for insomnia (ICBT-i) improves comorbid anxiety and depression-a meta-analysis of randomized controlled trials. PLoS One.

[35] Damghanian M, Alijanzadeh M (2018). Theory of planned behavior, self-stigma, and perceived barriers explains the behavior of seeking mental health services for people at risk of affective disorders. Soc Health Behav.

[36] Knowlden AP, Sharma M, Bernard AL (2012). A theory of planned behavior research model for predicting the sleep intentions and behaviors of undergraduate college students. J Prim Prev.

[37] Lao HC, Tao VY, Wu AM (2015). Theory of planned behaviour and healthy sleep of college students. Aust J Psychol.

[38] Strong C, Lin C, Jalilolghadr S, Updegraff JA, Broström A, Pakpour AH (2017). Sleep hygiene behaviours in Iranian adolescents: an application of the Theory of Planned Behavior. J Sleep Res.

[39] Walsh JJ, da Fonseca RS, Banta A (2005). Watching and participating in exercise videos: a test of the theory of planned behaviour, conscientiousness, and the role of implementation intentions. Psychol Health.

[40] Carver CS, Scheier MF (1982). Control theory: a useful conceptual framework for personality-social, clinical, and health psychology. Psychol Bull.

[41] Linardon J, Cuijpers P, Carlbring P, Messer M, Fuller-Tyszkiewicz M (2019). The efficacy of app-supported smartphone interventions for mental health problems: a meta-analysis of randomized controlled trials. World Psychiatry.

[42] Zachariae R, Lyby MS, Ritterband LM, O'Toole MS (2016). Efficacy of internet-delivered cognitive-behavioral therapy for insomnia: a systematic review and meta-analysis of randomized controlled trials. Sleep Med Rev.

[43] Andersson G, Carlbring P (2017). Internet-assisted cognitive behavioral therapy. Psychiatr Clin North Am.

[44] Choi YK, Demiris G, Lin S, Iribarren SJ, Landis CA, Thompson HJ, McCurry SM, Heitkemper MM, Ward TM (2018). Smartphone applications to support sleep self-management: review and evaluation. J Clin Sleep Med.

[45] Luik AI, van der Zweerde T, van Straten A, Lancee J (2019). Digital delivery of cognitive behavioral therapy for insomnia. Curr Psychiatry Rep.

[46] Kuhn E, Weiss BJ, Taylor KL, Hoffman JE, Ramsey KM, Manber R, Gehrman P, Crowley JJ, Ruzek JI, Trockel M (2016). CBT-I Coach: a description and clinician perceptions of a mobile app for cognitive behavioral therapy for insomnia. J Clin Sleep Med.

[47] Mead MP, Irish LA (2019). Application of health behaviour theory to sleep health improvement. J Sleep Res.

[48] American Psychiatric Association (2017). Diagnostic and statistical manual of mental disorders: DSM-5.

[49] Morin CM, Belleville G, Bélanger L, Ivers H (2011). The Insomnia Severity Index: psychometric indicators to detect insomnia cases and evaluate treatment response. Sleep.

[50] Lancee J, van den Bout J, Sorbi MJ, van Straten A (2013). Motivational support provided via email improves the effectiveness of internet-delivered self-help treatment for insomnia: a randomized trial. Behav Res Ther.

[51] Michie S, Richardson M, Johnston M, Abraham C, Francis J, Hardeman W, Eccles MP, Cane J, Wood CE (2013). The behavior change technique taxonomy (v1) of 93 hierarchically clustered techniques: building an international consensus for the reporting of behavior change interventions. Ann Behav Med.

[52] Yazdi Z, Sadeghniiat-Haghighi K, Zohal MA, Elmizadeh K (2012). Validity and reliability of the Iranian version of the insomnia severity index. Malays J Med Sci.

[53] Farrahi Moghaddam J, Nakhaee N, Sheibani V, Garrusi B, Amirkafi A (2012). Reliability and validity of the Persian version of the Pittsburgh Sleep Quality Index (PSQI-P). Sleep Breath.

[54] Pakpour AH, Hidarnia A, Hajizadeh E, Plotnikoff RC (2012). Action and coping planning with regard to dental brushing among Iranian adolescents. Psychol Health Med.

[55] Pakpour AH, Zeidi IM, Chatzisarantis N, Molsted S, Harrison AP, Plotnikoff RC (2011). Effects of action planning and coping planning within the theory of planned behaviour: a physical activity study of patients undergoing haemodialysis. Psychol Sport Exerc.

[56] Lin C, Yaseri M, Pakpour AH, Malm D, Broström A, Fridlund B, Burri A, Webb TL (2017). Can a multifaceted intervention including motivational interviewing improve medication adherence, quality of life, and mortality rates in older patients undergoing coronary artery bypass surgery? A multicenter, randomized controlled trial with 18-month follow-up. Drugs Aging.

[57] Lin C, Pakpour AH (2017). Using Hospital Anxiety and Depression Scale (HADS) on patients with epilepsy: confirmatory factor analysis and Rasch models. Seizure.

[58] Deng T, Wang Y, Sun M, Chen B (2013). Stage-matched intervention for adherence to CPAP in patients with obstructive sleep apnea: a randomized controlled trial. Sleep Breath.

