# Supplementary material for: Efficacy of a Theory-Based Cognitive Behavioral Technique App-Based Intervention for Patients With Insomnia: Randomized Controlled Trial
Source: J Med Internet Res. 2020 Apr 1;22(4):e15841. doi: 10.2196/15841 (PMC7160702; doi:10.2196/15841)

Multimedia Appendix 1. Behavior change techniques, content, and screenshots of app.

**Table S1.** Behavior Change Techniques (BCTs) included in the CBT-I App and their Targeted Outcomes

| Behavior change technique (BCT) | Examples of where BCTs are used in the CBT-I App | Targeted outcomes |
| --- | --- | --- |
|  |  |  |
| **BCT 5.3: Information about social and environmental consequences** |  |  |
|  | Week 1: Research suggests that adults need at least 7–8 hours of sleep each night to be well rested. More than one-third of adults’ report daytime sleepiness so severe that it interferes with work, driving, and social functioning at least a few days each month. | Attitude and intentions to perform sleep hygiene behaviors |
| **BCT 9.2: Pros and cons** |  |  |
|  | Week 1: The potential risks of insufficient sleep (e.g., blood pressure, stroke, diabetes and cardiovascular diseases).  The patients were asked to list the potential risks of poor sleep. e.g., ‘If I sleep enough daily, then I will have a healthier life’ | Attitude and intentions to perform sleep hygiene behaviors |
| **BCT 8.3: Habit formation** |  |  |
|  | Week 2: Go to bed and wake up at the same time each day. As creatures of habit, people have a hard time adjusting to changes in sleep patterns. Sleeping later at weekends does not fully make up for a lack of sleep during the week and often makes it harder to wake up early on Monday morning.  The patients were encouraged to make their bedroom more comfortable for sleeping through listening to their bodies, eating healthy foods, drinking safely and doing relaxation exercises before sleep.  Patients had to complete an exercise regarding writing down their new habits which they could adopt for this week. | Behavioral automaticity |
| **BCT 8.4: Habit reversal** |  |  |
|  | Week 3: Patients were asked try to imagine a place where they feel comfortable (e.g., a pleasant place) each night before bedtime | Behavioral automaticity |
| **BCT 12.1: Reconstructing the physical environment** |  |  |
|  | Week 2: Performing essential task to make bedroom more comfortable for sleeping.  e.g., Keep the temperature in your bedroom comfortable | Perceived behavioral control |
| **BCT 12.3: Reconstructing the social environment** |  |  |
|  | Week 2: Advice to minimize time spent on social media, with friends who drink or smoke heavily. | Perceived behavioral control |
| **BCT 1.4: Action planning** |  |  |
|  | Week 5: The patients were asked to create two plans specifying what (sleep hygiene behaviors), when (day), and where (place) they sleep | Action planning |
| **BCT 1.2: Problem-solving** |  |  |
|  | Week 5: The patients were asked to identify barriers that they might encounter when trying to sleep and generate strategies to overcome them.  e.g., ‘If I have an important meeting for the next day so I cannot sleep, then I will take a warm bath or relaxation exercises’ | Coping planning |
| **BCT 2.3: Self-monitoring of behavior** |  |  |
|  | Week 4: Patients were asked to monitor their sleep efficiency using a sleep chart. | Self-monitoring |
| ^a^ The BCTs are sourced from Michie et al.’s (2013) taxonomy | | |

**Table S2.** CBT-I content across the six weeks

| Week | Content |
| --- | --- |
|  |  |
| **1** |  |
|  | Participants in the intervention group received information about the importance of sleep, the amount of sleep needed, the main causes of insomnia, short-term and long-term complications and outcomes of insufficient sleep, and insomnia treatments (e.g., CBT-i and taking medications), the prevalence of insomnia in Iran. Moreover, and information concerning brain waves during sleep stages. Additionally, participants were asked to list the potential costs of poor sleep and the potential benefits of good sleep. At the end of the first week, the participants were encouraged to record sleeping information (sleep time, wake-up time, and time spent being in bed) using a notebook (which is incorporated in the app’s front page for easier and more accessible recording). |
| **2** |  |
|  | Week 2 focused on developing good health habits for sleep. The participants were taught to think about negative habits which can disrupt sleep [e.g., smoking at night, drinking tea, coffee, and/or alcohol, the bedroom environment [e.g., a dark, calm, and cool bedroom] and excessive worrying]. The participants were encouraged to change these sleep habits by reading a list of tips for making the bedroom more comfortable for sleeping. For example, listening to their bodies, eating healthy foods, drinking safely, and engaging in relaxation exercises before sleep. Participants were asked to write down their new habits which they can adopt for this week as a practice. Participants were asked to list the habits that affect their sleep and which they are trying to change (e.g., the bedroom, food, drinks) as next practice. At the end of this week, relaxation and meditation exercises were provided both in text and audio, along with the music tracks of these exercises. Participants were asked to perform these exercises every night before going to bed in comfortable places (for example, in bed) ensuring no disturbance (e.g., setting their smartphones to silent mode). |
| **3** |  |
|  | Relaxation practice was continued. Participants were asked trying to imagine a place where they feel comfortable (e.g., a pleasant place) each night before bedtime. In addition, participants were taught how to associate the bedroom only with sleep. This was done by asking participants to limit the amount of time that they spend in bed. To properly do this exercise, participants were asked to calculate the amount of time that they are awake at night using a ‘sleep efficiency chart’. Participants were asked to monitor their sleep performance using the sleep chart and/or check their sleep notes. At the end of each week, they were able to see their sleeping efficiency on the sleep chart (e.g., seeing if their sleep is increased by limiting the spent time in bed). |
| **4** |  |
|  | At the start of Week 4, participants were asked to monitor their sleep efficiency using the sleep chart to ensure that if they benefiting as intended from the third week exercises. The aim of Week 4 was to change misconceptions concerning sleep. A list of common misconceptions about sleep that individuals with insomnia experience was provided along with correct information. Differences between thoughts and feeling were reviewed. Some unhelpful thought patterns for sleeping were presented to the participants as examples. In addition, the participants were encouraged to remember their last time efforts for sleeping and to write down situation, thoughts, feelings, and behaviors in a table to help them to identify unhelpful thoughts. |
| **5** |  |
|  | At the start of Week 5, the participants were asked to refer to their sleep chart and check their sleep efficiency. In addition, the participants were asked to write down two situations in which they had good or bad sleep in the past week and what they wrote, as well as their thoughts, feelings, and behaviors that accompanied the situations. Participants were then asked to create plans specifying what (sleep hygiene behaviors), when (day), and where (place) they sleep. They were asked to write this information down in a table. The app reminded participants of their plans according to their recorded sleeping information (sleep time, wake up time, and time spent being in the bed) during past nights. In addition, participants were encouraged to identify barriers that they might encounter when trying to sleep and generate strategies to overcome them. |
| **6** |  |
|  | Participants were helped to learn how to relate bed with sleep and not awakening. More specifically they were reminded not to spend too much time awake in bed. All of the aforementioned techniques (i.e., sleep restriction), relaxation, sleep education, cognitive restructuring, action planning and coping planning) were summarized for the participants. During Week 6, paradoxical intention was used in which the participants were asked sleep for fewer hours than normal for one night to show that sleep deprivation does not necessarily affect daily living activities. At the end of Week 6, the participants were asked to complete a sleep diary form every day. |

^a^CBT-I=cognitive-behavioral therapy for insomnia

**Figure S1.** Screenshots of the app


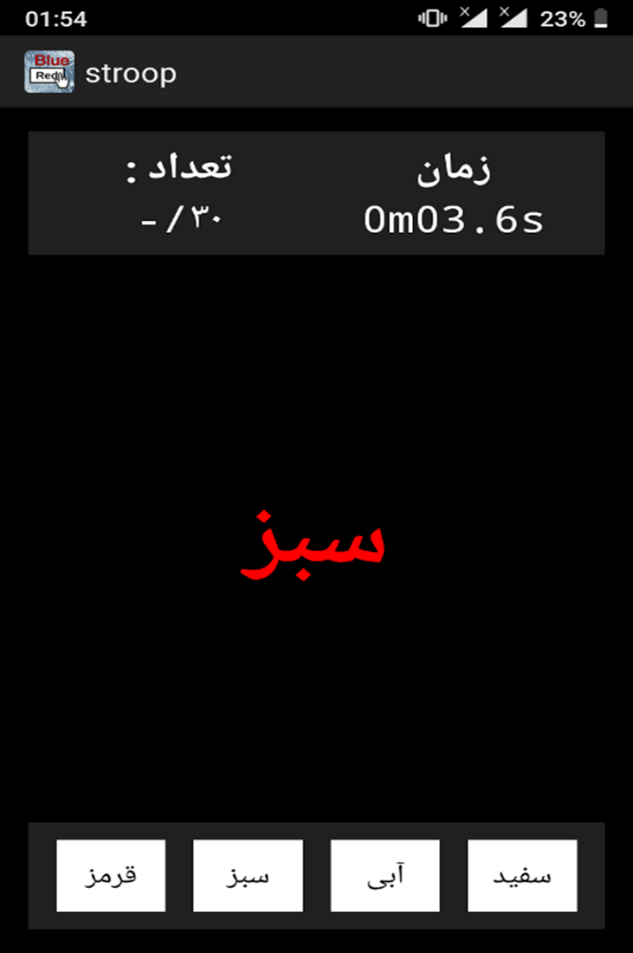

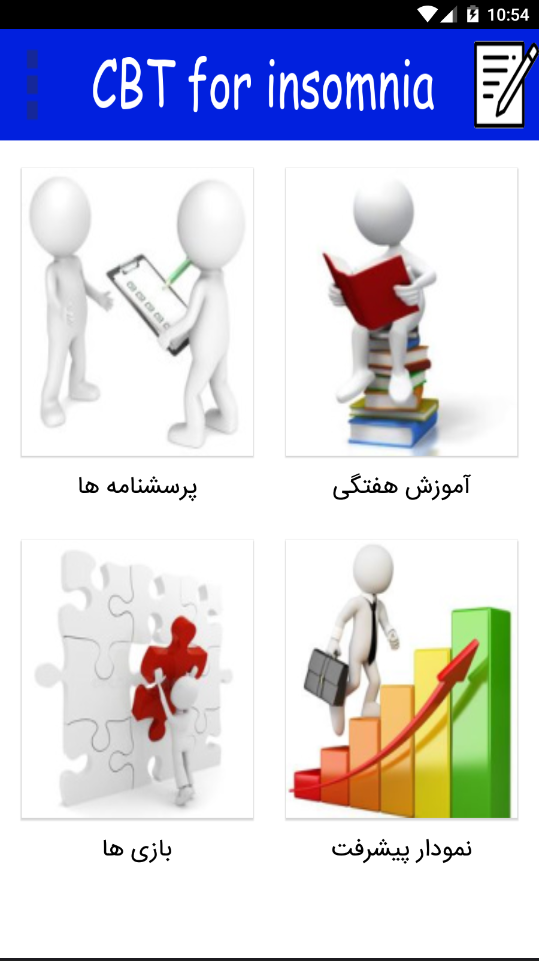

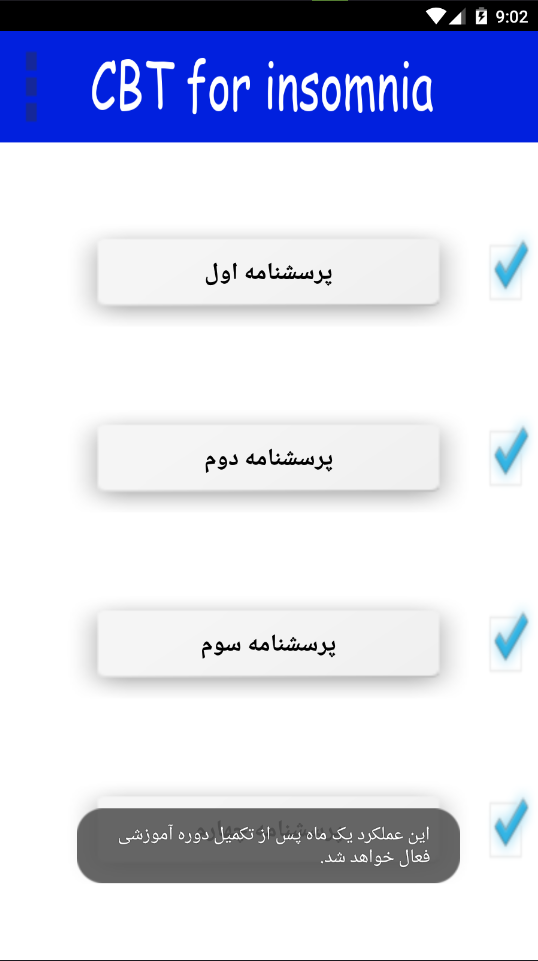

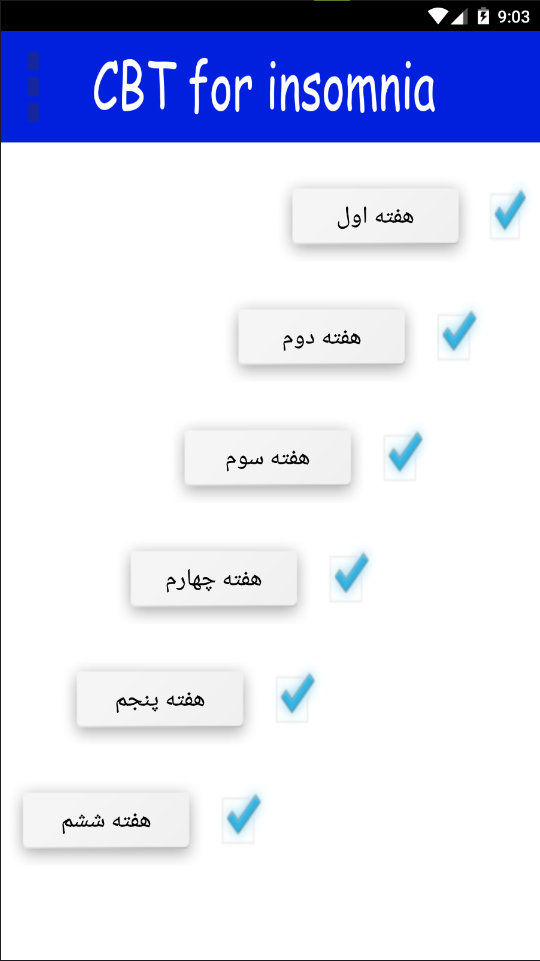

Supplement: Multimedia Appendix 1 [file jmir_v22i4e15841_app1.docx]
