# Supplementary material for: Efficacy of a Theory-Based Cognitive Behavioral Technique App-Based Intervention for Patients With Insomnia: Randomized Controlled Trial
Source: J Med Internet Res. 2020 Apr 1;22(4):e15841. doi: 10.2196/15841 (PMC7160702; doi:10.2196/15841)
Supplement: Multimedia Appendix 2 [file jmir_v22i4e15841_app2.docx]

Multimedia Appendix 2. Questionnaires used to assess primary and secondary outcomes.

**Questionnaire items in Attitude**

**On each line below and between each extreme, place a slash closest to your first impression:**

To make my bedroom/sleep environment restful would be…?

| 5 | 4 | 3 | 2 | 1 |
| --- | --- | --- | --- | --- |
| *pleasant* |  |  |  | *unpleasant* |
| *Bad* |  |  |  | *Good* |
| *Foolish* |  |  |  | *Wise* |
| *Correct* |  |  |  | *Incorrect* |
| *enjoyable* |  |  |  | *unenjoyable* |
| *unsatisfying* |  |  |  | *satisfying* |
| *useful* |  |  |  | *useless* |

To avoid going to bed feeling hungry or thirsty would be…?

| 5 | 4 | 3 | 2 | 1 |
| --- | --- | --- | --- | --- |
| *Bad* |  |  |  | *Good* |
| *Correct* |  |  |  | *Incorrect* |
| *useful* |  |  |  | *useless* |

To avoid anxiety and stress-provoking activity before bed would be…?

| 5 | 4 | 3 | 2 | 1 |
| --- | --- | --- | --- | --- |
| *Foolish* |  |  |  | *Wise* |
| *Correct* |  |  |  | *Incorrect* |

**Questionnaire items in Perceived Behavioral Control**

I am confident that every day I can prevent anxiety-provoking activity before bedtime

| Totally agree | Agree | Neither agree  nor disagree | Disagree | Totally disagree |
| --- | --- | --- | --- | --- |
| 5 | 4 | 3 | 2 | 1 |

I am confident that every day I can prevent going to bed feeling hungry or thirsty before bedtime

| Totally agree | Agree | Neither agree  nor disagree | Disagree | Totally disagree |
| --- | --- | --- | --- | --- |
| 5 | 4 | 3 | 2 | 1 |

I am confident that every day I can make my bedroom/sleep environment restful before bedtime

| Totally agree | Agree | Neither agree  nor disagree | Disagree | Totally disagree |
| --- | --- | --- | --- | --- |
| 5 | 4 | 3 | 2 | 1 |

**Questionnaire items in Behavioral Intention**

Over the next week, I intend to make my bedroom restful

| Totally agree | Agree | Neither agree  nor disagree | Disagree | Totally disagree |
| --- | --- | --- | --- | --- |
| 5 | 4 | 3 | 2 | 1 |

Over the next week, I plan to make my bedroom restful

| Totally agree | Agree | Neither agree  nor disagree | Disagree | Totally disagree |
| --- | --- | --- | --- | --- |
| 5 | 4 | 3 | 2 | 1 |

Over the next week, I intend to prevent going to bed feeling hungry or thirsty

| Totally agree | Agree | Neither agree  nor disagree | Disagree | Totally disagree |
| --- | --- | --- | --- | --- |
| 5 | 4 | 3 | 2 | 1 |

Over the next week, I plan to prevent going to bed feeling hungry or thirsty

| Totally agree | Agree | Neither agree  nor disagree | Disagree | Totally disagree |
| --- | --- | --- | --- | --- |
| 5 | 4 | 3 | 2 | 1 |

Over the next week, I intend to avoid anxiety and stress-provoking activity before bed

| Totally agree | Agree | Neither agree  nor disagree | Disagree | Totally disagree |
| --- | --- | --- | --- | --- |
| 5 | 4 | 3 | 2 | 1 |

Over the next week, I plan to avoid anxiety and stress-provoking activity before bed

| Totally agree | Agree | Neither agree  nor disagree | Disagree | Totally disagree |
| --- | --- | --- | --- | --- |
| 5 | 4 | 3 | 2 | 1 |

**Questionnaire items in Action Planning**

**I have made a detailed plan regarding when to perform sleep hygiene behaviors over the next six months.**

| Totally agree | Agree | Neither agree  nor disagree | Disagree | Totally disagree |
| --- | --- | --- | --- | --- |
| 5 | 4 | 3 | 2 | 1 |

**I have made a detailed plan regarding where to perform sleep hygiene behaviors over the next six months.**

| Totally agree | Agree | Neither agree  nor disagree | Disagree | Totally disagree |
| --- | --- | --- | --- | --- |
| 5 | 4 | 3 | 2 | 1 |

**I have made a detailed plan regarding how to perform sleep hygiene behaviors over the next six months.**

| Totally agree | Agree | Neither agree  nor disagree | Disagree | Totally disagree |
| --- | --- | --- | --- | --- |
| 5 | 4 | 3 | 2 | 1 |

**I have made a detailed plan regarding how often to perform sleep hygiene behaviors over the next six months.**

| Totally agree | Agree | Neither agree  nor disagree | Disagree | Totally disagree |
| --- | --- | --- | --- | --- |
| 5 | 4 | 3 | 2 | 1 |

**Questionnaire items in Coping Planning**

**I have made a detailed plan regarding what to do if something interferes with my plans**

| Totally agree | Agree | Neither agree  nor disagree | Disagree | Totally disagree |
| --- | --- | --- | --- | --- |
| 5 | 4 | 3 | 2 | 1 |

**I have made a detailed plan regarding how to cope with possible setbacks**

| Totally agree | Agree | Neither agree  nor disagree | Disagree | Totally disagree |
| --- | --- | --- | --- | --- |
| 5 | 4 | 3 | 2 | 1 |

**I have made a detailed plan regarding what to do in difficult situations to act according to my intentions**

| Totally agree | Agree | Neither agree  nor disagree | Disagree | Totally disagree |
| --- | --- | --- | --- | --- |
| 5 | 4 | 3 | 2 | 1 |

**I have made a detailed plan regarding how to motivate myself**

| Totally agree | Agree | Neither agree  nor disagree | Disagree | Totally disagree |
| --- | --- | --- | --- | --- |
| 5 | 4 | 3 | 2 | 1 |

**I have made a detailed plan regarding which good opportunities for action to take**

| Always | Very often | Sometimes | Seldom | Never |
| --- | --- | --- | --- | --- |
| 5 | 4 | 3 | 2 | 1 |

**Questionnaire items in Self-monitoring**

**I keep track of how much time I spend sleeping**

| Totally agree | Agree | Neither agree  nor disagree | Disagree | Totally disagree |
| --- | --- | --- | --- | --- |
| 5 | 4 | 3 | 2 | 1 |

**I pay attention to how tired or rested I feel each day**

| Totally agree | Agree | Neither agree  nor disagree | Disagree | Totally disagree |
| --- | --- | --- | --- | --- |
| 5 | 4 | 3 | 2 | 1 |

**I take care to note the time that I go to bed and wake each day**

| Totally agree | Agree | Neither agree  nor disagree | Disagree | Totally disagree |
| --- | --- | --- | --- | --- |
| 5 | 4 | 3 | 2 | 1 |

**Questionnaire items in Behavioral Automaticity**

**Sleep hygiene behavior is something…**

**I do automatically**

| Totally agree | Agree | Neither agree  nor disagree | Disagree | Totally disagree |
| --- | --- | --- | --- | --- |
| 5 | 4 | 3 | 2 | 1 |

**I do without having to consciously remember**

| Totally agree | Agree | Neither agree  nor disagree | Disagree | Totally disagree |
| --- | --- | --- | --- | --- |
| 5 | 4 | 3 | 2 | 1 |

**I do without thinking**

| Totally agree | Agree | Neither agree  nor disagree | Disagree | Totally disagree |
| --- | --- | --- | --- | --- |
| 5 | 4 | 3 | 2 | 1 |

**I start doing before I realize I’m doing it**

| Totally agree | Agree | Neither agree  nor disagree | Disagree | Totally disagree |
| --- | --- | --- | --- | --- |
| 5 | 4 | 3 | 2 | 1 |

**Questionnaire items in Sleep Hygiene Behaviors**

**How many days did you make your bedroom restful over the past week?**

| 7 | 6 | 5 | 4 | 3 | 2 | 1 | 0 |
| --- | --- | --- | --- | --- | --- | --- | --- |

**How many days did you avoided going to bed feeling hungry or thirsty over the past week?**

| 7 | 6 | 5 | 4 | 3 | 2 | 1 | 0 |
| --- | --- | --- | --- | --- | --- | --- | --- |

**How many days did you avoided anxiety and stress-provoking activity before bed over the past week?**

| 7 | 6 | 5 | 4 | 3 | 2 | 1 | 0 |
| --- | --- | --- | --- | --- | --- | --- | --- |

**Questionnaire items in Insomnia Severity Index (ISI)**

Please rate the CURRENT (i.e. LAST 2 WEEKS) SEVERITY of your insomnia problem(s).

| Insomnia Problem | None | Mild | Moderate | Severe | Very Severe |
| --- | --- | --- | --- | --- | --- |
| 1.Difficulty falling asleep | 0 | 1 | 2 | 3 | 4 |
| 2.Difficulty staying asleep | 0 | 1 | 2 | 3 | 4 |
| 3. Problems waking up too early | 0 | 1 | 2 | 3 | 4 |

4. How SATISFIED/DISSATISFIED are you with your CURRENT sleep pattern?

| Very Dissatisfied | Dissatisfied | Moderately Satisfied | Satisfied | Very Satisfied |
| --- | --- | --- | --- | --- |
| 4 | 3 | 2 | 1 | 0 |

5. How NOTICEABLE to others do you think your sleep problem is in terms of impairing the quality of your life?

| Very Much Noticeable | Much | Somewhat | A Little | Not at all Noticeable |
| --- | --- | --- | --- | --- |
| 4 | 3 | 2 | 1 | 0 |

6. How WORRIED/DISTRESSED are you about your current sleep problem?

| Very Much Worried | Much | Somewhat | A Little | Not at all Worried |
| --- | --- | --- | --- | --- |
| 4 | 3 | 2 | 1 | 0 |

7. To what extent do you consider your sleep problem to INTERFERE with your daily functioning (e.g. daytime fatigue, mood, ability to function at work/daily chores, concentration, memory, mood, etc.) CURRENTLY?

| Very Much Interfering | Much | Somewhat | A Little | Not at all Interfering |
| --- | --- | --- | --- | --- |
| 4 | 3 | 2 | 1 | 0 |

**Questionnaire items in Pittsburgh Sleep Quality Index (PSQI)**

1. When have you usually gone to bed? ______________

2. How long (in minutes) has it taken you to fall asleep each night? ______________

3. When have you usually gotten up in the morning? ______________

4. How many hours of actual sleep do you get at night? (This may be different than the number of hours you spend in bed) ______________

| Three or a more times week(3) | Once or twice week(2) | Less than once a week(1) | Not during the past month(0) | 5. During the past month, how often have you had trouble sleeping because you… | 5 |
| --- | --- | --- | --- | --- | --- |
|  |  |  |  | a. Cannot get to sleep within 30 minutes  f. Feel too cold | 5.1 |
|  |  |  |  | b. Wake up in the middle of the night or early morning | 5.2 |
|  |  |  |  | c. Have to get up to use the bathroom | 5.3 |
|  |  |  |  | d. Cannot breathe comfortably | 5.4 |
|  |  |  |  | e. Cough or snore loudly | 5.5 |
|  |  |  |  | f. Feel too cold | 5.6 |
|  |  |  |  | g. Feel too hot | 5.7 |
|  |  |  |  | h. Have bad dreams | 5.8 |
|  |  |  |  | i. Have pain | 5.9 |
|  |  |  |  | j. Other reason(s), please describe, including how often you have had trouble sleeping because of this reason(s): | 5.10 |
|  |  |  |  | During the past month, how often have you taken medicine (prescribed or “over the counter”) to help you sleep? | 6 |
|  |  |  |  | During the past month, how often have you had trouble staying awake while driving, eating meals, or engaging in social activity? | 7 |
|  |  |  |  | During the past month, how much of a problem has it been for you to keep up enthusiasm to get things done? | 8 |
| Very bad (3) | Fairly bad (2) | Fairly good(1) | Very good (0) | 9. During the past month, how would you rate your sleep quality overall? | 9 |
|  |  |  |  |  |  |

**Questionnaire items in Hospital Anxiety and Depression Scale (HADS)**

| I feel tense or 'wound up':  3 Most of the time  2 A lot of the time  1 From time to time, occasionally  0 Not at all | I feel as if I am slowed down:  3 Nearly all the time  2 Very often  1 Sometimes  0 Not at all |
| --- | --- |
| I still enjoy the thing I used to enjoy:  0 Definitely as much  1 Not quite so much  2 Only a little  3 Hardly at all | I get a sort of frightened feeling like 'butterflies' in the stomach:  0 Not at all  1 Occasionally  2 Quite Often  3 Very Often |
| I get a sort of frightened feeling as if something awful is about to happen:  3 Very definitely and quite badly  2 Yes, but not too badly should  1 A little, but it doesn't worry me  0 Not at all | I have lost interest in my appearance:  3 Definitely  2 I don't take as much care as I  1 I may not take quite as much care  0 I take just as much care as ever |
| I can laugh and see the funny side of things:  0 As much as I always could  1 Not quite so much now  2 Definitely not so much now  3 Not at all | I feel restless as I have to be on the move:  3 Very much indeed  2 Quite a lot  1 Not very much  0 Not at all |
| Worrying thoughts go through my mind:  3 A great deal of the time  2 A lot of the time  1 From time to time, but not too often  0 Only occasionally | look forward with enjoyment to things:  0 As much as I ever did  1 Rather less than I used to  2 Definitely less than I used to  3 Hardly at all |
| I feel cheerful:  3 Not at all  2 Not often  1 Sometimes  0 Most of the time | I get sudden feelings of panic:  3 Very often indeed  2 Quite often  1 Not very often  0 Not at all |
| I can sit at ease and feel relaxed:  0 Definitely  1 Usually  2 Not Often  3 Not at all | I can enjoy a good book or radio or TV program:  0 Often  1 Sometimes  2 Not often  3 Very seldom |
